# Chitosan gel vaccine protects against tumour growth in an intracaecal mouse model of cancer by modulating systemic immune responses

**DOI:** 10.1186/s12865-016-0178-4

**Published:** 2016-10-18

**Authors:** Andrew J. Highton, Adam Girardin, Georgia M. Bell, Sarah M. Hook, Roslyn A. Kemp

**Affiliations:** 1 Department of Microbiology and Immunology, Otago School of Medical Sciences, University of Otago, PO Box 56, Dunedin, 9054 New Zealand; 2 School of Pharmacy, University of Otago, Dunedin, New Zealand

**Keywords:** Colon cancer, IFN-γ, T cells, Vaccination, Tumour, Caecum

## Abstract

**Background:**

Vaccination generating a robust memory population of CD8^+^ T cells may provide protection against cancer. However, immune therapies for cancer are influenced by the local tumour immune microenvironment. An infiltrate of T cells into tumours of people with colorectal cancer has proven to be a significant indicator of good prognosis.

**Methods:**

We used an intracaecal mouse model of cancer to determine whether a protective immune response against a mucosal gut tumour could be generated using a systemic intervention. We investigated the generation of murine memory CD8^+^ T cells using a sustained antigen release vaccine vehicle (chitosan gel; Gel + OVA) containing the model antigen ovalbumin, chitosan gel alone (Gel) or conventional dendritic cell vaccination (DC + OVA) using the same protein antigen.

**Results:**

Following vaccination with Gel + OVA, CD8^+^ T cell memory populations specific for ovalbumin protein were detected. Only vaccination with Gel + OVA gave decreased tumour burden compared to unvaccinated or DC + OVA-vaccinated mice in the intracaecal cancer challenge model.

**Conclusion:**

These results indicate that subcutaneous vaccination with Gel + OVA generates a population of functional CD8^+^ memory T cells in lymphoid tissue able to protect against intracaecal tumour challenge. Vaccination with chitosan gel may be valuable in anti-cancer treatment at both peripheral and mucosal sites.

**Electronic supplementary material:**

The online version of this article (doi:10.1186/s12865-016-0178-4) contains supplementary material, which is available to authorized users.

## Background

Colorectal cancer (CRC) is the fourth leading cause of cancer related death worldwide [1]. In most cases, the disease occurs sporadically but can also be preceded by inflammatory bowel disease or a familial genetic predisposition, for example, familial adenomatous polyposis (FAP) [2, 3]. As with other cancers, the immune system plays a large role in the control of disease. Work by Galon et al has shown that a large infiltrating population of CD8^+^ T cells predicts improved patient outcome [4]. More mixed results have been obtained regarding other immune cell populations in tumour tissue and their relation to patient outcome [5–8].

The gut is an immunosuppressive environment and control of local disease may also be altered. For example, in many cancers an increased frequency of infiltrating regulatory T cells (Tregs) correlates with poor outcome but in colorectal cancer, the infiltrate of Tregs has been associated with both good and poor outcomes [7]. It is therefore important to consider this altered environment and to perform observations at this distinct site when considering tissue targeted immunotherapies. Orthotopic models of colorectal cancer have been described [9–14], but we wished to investigate the ability to modulate the *systemic* immune response to prevent local tumour growth in the gut.

Previously, we showed that vaccination with chitosan gel could generate a population of CD8^+^ memory T cells in both peripheral and gut-associated lymphoid sites [15]. Furthermore, vaccination with chitosan gel also provided protection in a subcutaneous tumour challenge model, both prophylactically and therapeutically [15]. However due to the specialised nature of the gut immune system the ability of systemic immunisation to protect against a gut tumour is unknown. We showed that vaccination with chitosan gel was protective in an intracaecal mouse model of cancer, while vaccination with DCs was not. The gel-mediated protection was associated with an increase in antigen specific T cells and T cells producing IFN-γ.

## Methods

### Mice

C57BL/6 and OT-I transgenic mice were obtained from the HTRU (University of Otago, Dunedin, NZ) and were bred and housed under specific pathogen free conditions. All experimental procedures were approved by the University of Otago Animal Ethics Committee. No changes in weight or general health (positive or negative) were observed in mice following tumour injection with or without vaccination.

### Cell lines

B16-OVA and B16-*luc* cell lines were cultured in complete RPMI (with 100 μg/mL Penicillin, 100 μg/mL Streptomycin, 55 μM 2-mercaptoethanol, (all from Invitrogen, Carlsbad, USA), 5 % fetal calf serum (PAA laboratories, Morningside, QLD, AU) at 37 °C, 5 % CO_2_. B16-OVA cells were grown in 5 % complete RPMI with the addition of geneticin (Invitrogen) at 500 μg/mL to prevent loss of ovalbumin protein expression.

### Formulation of chitosan hydrogel

Chitosan (1 % w/v) (Sigma-Aldrich-Aldrich, St Louis, MO, USA) and methylcellulose (0.5 % w/v) (Sigma-Aldrich) were added to 0.05 mol/L hydrochloric acid (VWR, Radnor, PA, USA) and stirred at 4 °C overnight. Glycerol 2-phosphate disodium hydrate (Sigma-Aldrich) was added drop wise to give the solution thermosensitive properties and stirred for a further hour. Ovalbumin protein (OVA; Sigma-Aldrich) and Quil-A (QA; Brenntag Biosector, Denmark) were added for a final concentration of 100 μg/mL and 200 μg/mL, respectively, then stirred for a further 30 min to ensure uniform distribution in solution.

### Bone marrow derived dendritic cell generation

Bone marrow harvested from the leg bones of naïve C57BL/6 mice was cultured for 7 days in complete RPMI and 20 ng/mL GM-CSF (MyBiosource, San Diego, CA, USA) as described [15]. For OVA vaccinations, cells were pulsed with OVA protein overnight on day 5 at a final concentration of 200 μg/mL followed by 1 μg/mL of lipopolysaccharide (Sigma-Aldrich) overnight on day 6. Dendritic cells were MHCII^+^ and CD80^hi^.

### Vaccination and adoptive transfer of cells

Two hundred microlitres of chitosan hydrogel containing both OVA and QA (Gel + OVA) or QA alone (Gel), or 2 × 10^5^ OVA-pulsed DCs (DC + OVA) in 200 μl phosphate buffered saline (PBS; NaCl – Biolab, Australia, 137 mM; KCl – VWR, 2.7 mM; Na_2_HPO_4_ – VWR, 4.3 mM; KH_2_PO_4_ – Merck, 1.4 mM), were injected subcutaneously into the flank of C57BL/6 mice. 2 × 10^5^ naïve OT-I lymphocytes [16] were injected intravenously in 200 μl of PBS into the tail vein of mice at the time of vaccination.

### Subcutaneous and intracaecal tumour challenge

Mice were injected with 2 × 10^5^ B16-OVA or B16-*luc* cells subcutaneously in the flank in 100 μL PBS 30 days following vaccination. Surgery for intracaecal injection was carried out according to Tseng et al [17]. Briefly, mice were anaesthetised one at a time using a combination of ketamine, domitor and atropine injected subcutaneously. Pre-operative carporfen was also administered subcutaneously. The abdomen of the mouse was shaved and oil was applied to the eyes. An incision was made through the skin and peritoneum. The caecum was externalised and 25 μL PBS containing 1×10^5^ cells was injected under a 10x surgical microscope into the subserosa of the caecum. The caecum was repositioned in the abdominal cavity and peritoneum and skin were resutured separately. The mouse was injected with amphoprim antibiotic and antisedan to reverse the anaesthetic effect. Mice were monitored twice daily for 5 days to assess recovery and carporfen and amphoprim were administered twice daily for 2–3 days. All drugs and anaesthetics were distributed by the Animal Welfare Office, University of Otago.

### Bioluminescence

Mice were injected intraperitoneally with 200 μL of luciferin (Pure Science, Porirua, New Zealand) and placed in the induction chamber of an isofluorane based gas anaesthetic device. At 5 min following administration of luciferin, mice were x-rayed for 30 s and imaged to detect bioluminescence for 5 min. Bruker MI (Bruker, Billerica, MA, USA) and ImageJ (NIH, Bethesda, MD, USA) were used for image capture and analysis.

### Flow cytometry

Single cell suspensions were resuspended in 1 mL PBS and incubated with titrated concentrations of Live/Dead Fixable Red Dead Cell Stain (Invitrogen) for 30 min at 4 °C in the dark. Samples were washed with FACS buffer (PBS + 0.5 % Fetal Calf Serum and 0.01 % NaN_3_ – VWR, Radnor, PA, USA) and incubated with titrated concentrations of the following fluorescently labelled anti-mouse antibodies for 10 min at 4 °C in the dark: CD8-PerCPCy5.5 (53–6.7), CD45.1-BV421 (A20), CD122-PE (5H4; all from BioLegend, San Diego, CA, USA), CD4-APCH7 (GK1.5), CD19-APCH7 (1D3), Vα2-APC (B20.1), Vβ5-FITC (MR9-4), CD44-V500 (1 M7; all from BD Biosciences, Franklin Lakes, NJ, USA). Following this, samples were washed in FACS buffer, fixed in 1 % paraformaldehyde (Sigma-Aldrich) for 30 min then resuspended in FACS buffer for acquisition on an LSR Fortessa (BD Biosciences) and analysed using FlowJo (Treestar, Ashland, OR, USA). For cytokine detection cells were restimulated with phorbol-12-myristate-13-acetate (PMA) and ionomycin, and brefeldin-A (all from Sigma-Aldrich) was added to samples 2 h before harvesting. Samples were incubated with IFN-γ-PE (XMG1.2; BioLegend) antibodies in permeabilisation buffer for 30 min in the dark at 4 °C and washed three times in permeabilisation buffer.

### Statistical analysis

GraphPad Prism (GraphPad, La Jolla, CA, USA) was used for all graphs and statistical analysis. Significance was calculated using one-way ANOVA and a Tukey post-hoc test as indicated in figure legends.

## Results

### Development of an intra-caecal model of cancer

Site-specific murine models of cancer are more reflective of human disease. We wished to develop a model that would allow us to make comparisons with human colorectal cancer, particularly in regard to local immune responses. Based on our previous studies showing protection against melanoma using a chitosan hydrogel vaccine delivery formulation, we chose to test immune responses to the vaccine against the same tumour cell line, but injected intra-caecally, to better reflect the local tumour environment of human colorectal cancer. Preliminary studies used a B16*-luc* cell line to establish the model, as it was easily detectable via bioluminescence in vitro and after subcutaneous injection in vivo. Similar growth kinetics were observed in the B16-OVA cell line as the B16-*luc* cell line, both in vitro and in vivo (data not shown). In orthotopic mouse models of colorectal cancer metastases are usually minimal, although they are a major component of the human disease. There is evidence that shows B16 cell lines may metastasise to lymph nodes in similar orthotopic models suggesting an advantage for the use of this cell line [18].

**Fig. 1:**
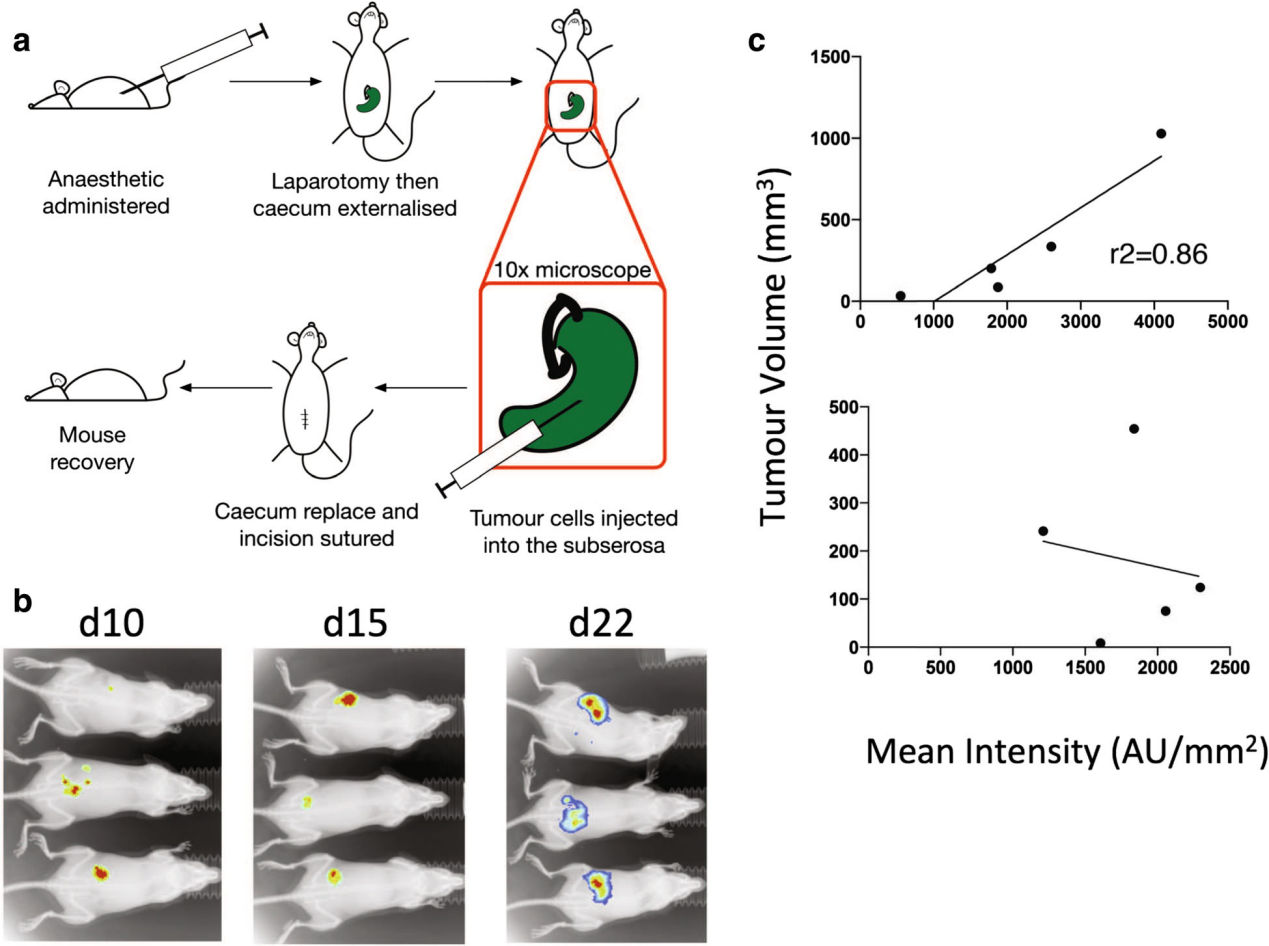
Intracaecal Model of Cancer **a** Mice were anaesthetised and a laparotomy performed. The caecum was exteriorised and tumour cells were injected into the caecal cell wall under 10× magnification. The caecum was returned to the abdominal cavity and the incision was sutured. **b** 1 × 10^5^ B16-*luc* cells were injected intracaecally as described. Luciferin was administered to mice and bioluminescence was detected 10, 15 and 22 days later. Data shows a single representative experiment of two. **c** 2 × 10^5^ B16-*luc* cells were injected subcutaneously (*top*) or intracaecally (*bottom*). Correlation of bioluminescent signal produced (arbitrary units/mm^2^) to tumour volume are shown. Data show a single representative experiment. Each experiment was performed twice

Mice were anaesthetised and the caecum was surgically exteriorised following laparotomy so that tumour cells could be injected into the caecal cell wall. Cells were injected into the subserosa of the caecum and mice were monitored during recovery and were imaged at days 10, 15 and 22 following surgery to detect the bioluminescent activity of growing tumour cells (Fig. 1a). At all time points, a bioluminescent signal could be detected in mice (Fig. 1b). This signal was localised to the predicted site of caecal injection and was later verified following euthanasia of mice and direct visualisation of tumours.

These results showed that the intra-caecal mouse model of cancer was viable. Mice recovered well following surgery, with no discernible weight loss, the B16-*luc* cell line was able to grow in the gut and the bioluminescent signal from the tumour could be detected even at this increased depth compared to subcutaneous tumours. However, B16-*luc* tumour size did not correlate well with the bioluminescent signal produced (Fig. 1c) and could not be used to quantify growth non-invasively. However the bioluminescent signal did allow for the detection of extensive tumour growth such that mice could be euthanised at an early humane endpoint. Subsequent experiments showed the B16-OVA cell line grew in vivo with similar kinetics and time to endpoint.

### Different immune infiltrate in intracaecal compared with subcutaneous tumour microenvironment

To determine whether the immune infiltration of cells into tumours differed depending on the location of the tumour, mice were injected with B16-*luc* expressing cells subcutaneously or intracaecally. Twenty days later, tumours and peripheral lymph nodes were excised and labelled with antibodies to surface markers to detect the phenotype of the immune cell infiltrate, including CD3^+^, CD4^+^ and CD8^+^ T cells, B cells, macrophages and dendritic cells (Additional file 1: Figure S1).

**Fig. 2:**
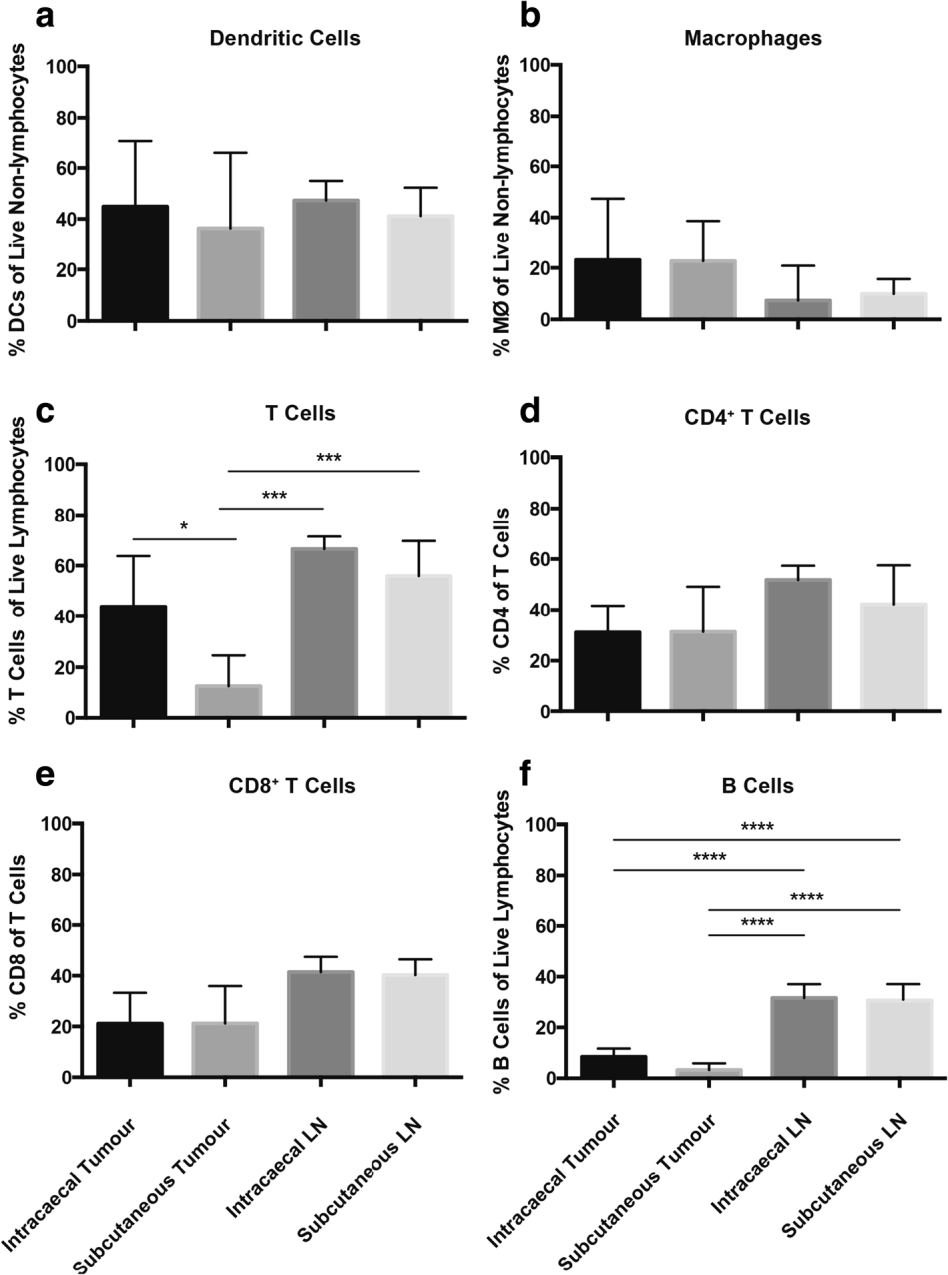
Immune infiltrate can be detected in mice challenged with B16-luc **a** Percentage CD11c^+^ of live non-lymphocytes (dendritic cells), **b** percentage F480^+^ of non-lymphocytes (macrophages), **c** percentage CD3^+^ of lymphocytes (T cells), **d** percentage CD4^+^ of T cells, **e** percentage CD8^+^ of T cells and **f** percentage CD19^+^ of lymphocytes (B cells) in tumours or draining lymph nodes. Data are shown as mean ± standard deviation, *n* = 4–5, representative of two individual experiments. One-way ANOVA with Turkey post-hoc test was performed. *P < 0.05, **P < 0.01, ***P < 0.001, ****P < 0.0001

Frequencies of populations of all assessed immune cell types in the lymph nodes were similar between mice receiving intracaecal or subcutaneous tumours (Fig. 2). However, within the tumour, immune cell infiltrates differed. No significant difference was seen in the frequencies of dendritic cells expressing CD11c and macrophages expressing F4/80 between intracaecal and subcutaneous tumours (Fig. 2a, b). A higher frequency of CD3^+^ T cells was present in the intracaecal tumours than the subcutaneous tumours (Fig. 2c). Interestingly, within the CD3^+^ population, there was no difference in the distribution of CD4^+^ and CD8^+^ T cells between intracaecal and subcutaneous tumours (Fig. 2d, e). This may be due to the presence of CD3^+^CD4^−^CD8^−^ T cell subsets, including MAIT cells [19] or double negative inflammatory cells [20]. A small but non-significant increase was seen in the frequency of B cells in intracaecal tumours compared to subcutaneous tumours (Fig. 2f). There was no significant difference in tumour size at the time of excision between intra-caecal and subcutaneous tumours (average size 210 mm^3^ and 143 mm^3^, respectively), in line with our previous data [15].

The differences in infiltrating lymphocytes between intracaecal and subcutaneous tumours demonstrate that this mouse model of cancer may provide additional insight into immune involvement in cancer of the colon.

### Chitosan gel vaccine is protective in a mouse model of colorectal cancer

We have previously demonstrated that subcutaneous injection with vaccine in a sustained release gel (chitosan gel) generated a population of CD8^+^ memory T cells at both peripheral lymphoid sites and gut-associated lymphoid sites [15]. Furthermore, this response protected against a subsequent subcutaneous tumour challenge and was effective in treating established tumours. Due to the presence of CD8^+^ memory T cells in gut-associated lymphoid tissues following vaccination, we hypothesised that vaccination with the sustained release gel would also provide protection against gut tumours. To test this hypothesis, the intra-caecal mouse model of cancer was used to challenge vaccinated mice.

Mice were vaccinated subcutaneously with vaccine (OVA and the adjuvant Quil A) in chitosan gel (Gel + OVA), with gel and Quil A but no antigen (Gel), with a control immune-based vaccine of activated DC pulsed with OVA (DC + OVA) or were unvaccinated. No change in weight, health or survival was observed following vaccination, consistent with previous results [15]. All mice received adoptive transfer of OT-I lymphocytes as described previously in order to accurately quantify antigen-specific memory responses [15]. Thirty to 34 days later, mice were injected intracaecally with B16-OVA. Mice were euthanised at the pre-determined time point of 3 weeks based on tumour growth determined in Fig. 1 (and data not shown); hence no survival data are available.

**Fig. 3:**
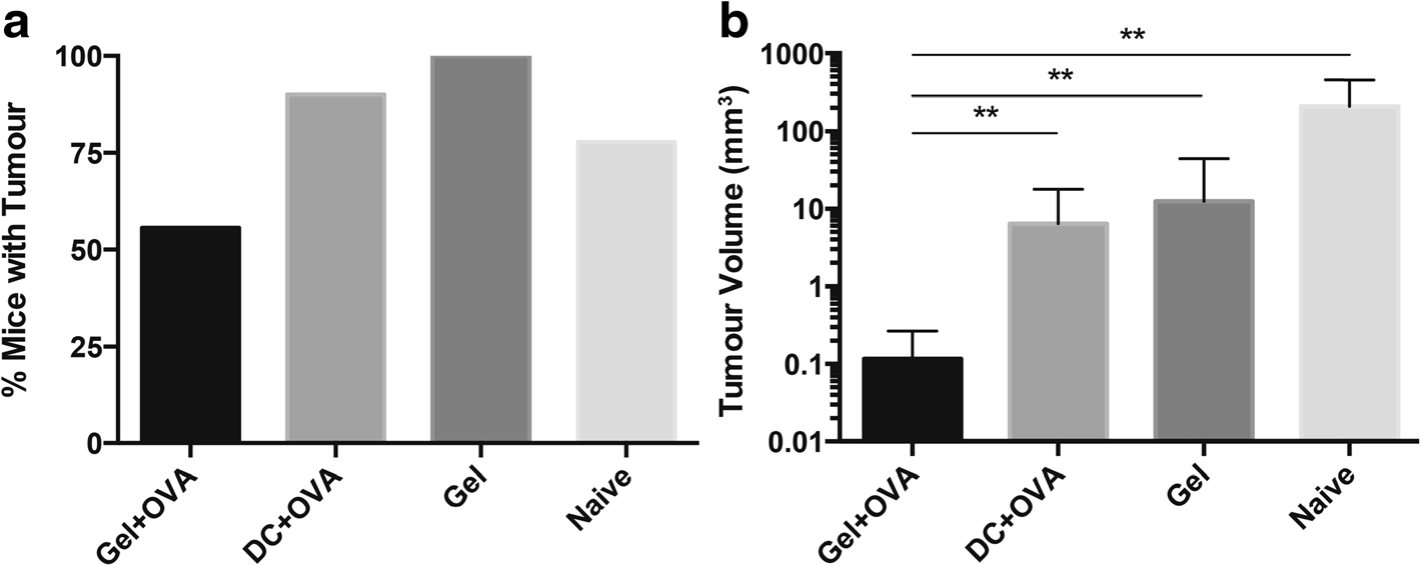
Vaccination with a sustained release gel reduces the frequency of tumour development. Mice received adoptive transfer of OT-I lymphocytes and were not vaccinated or were vaccinated with antigen in a chitosan gel (Gel + OVA), the gel alone (Gel) or with antigen pulsed-DC (DC + OVA) then challenged intracaecally with B16-OVA 30 days later. **a** Percentage of mice with visible tumours and **b** mean volume of tumours at day 21. Data are shown as mean ± standard deviation, Data pooled from two individual experiments, *n* = 8–9. One-way ANOVA with Tukey post-hoc test was performed. **P < 0.01

Tumours developed in all of the mice vaccinated with Gel, and in 90 % of mice vaccinated with DC + OVA. Only 50 % of the mice vaccinated with Gel + OVA developed tumours (Fig. 3a). Tumours in unimmunised mice and in mice immunised with the dendritic cell vaccine or with gel without antigen were larger than those vaccinated with gel plus antigen (Fig. 3b). These data indicate that vaccination with chitosan gel is superior to vaccination with a DC vaccine in this model.

### Chitosan gel vaccine generates antigen specific memory CD8^+^ T cells in gut-associated tissue

Fifty one to 54 days following vaccination with Gel + OVA, DC + OVA or Gel (21 days after intracaecal tumour challenge with B16-OVA cells) peripheral lymph nodes, spleens, mesenteric lymph nodes and Peyer’s patches were excised. The frequencies of antigen-specific (Vα2^+^Vβ5^+^) and memory T cells were examined (Additional file 2: Figure S2). As many tumours, particularly in mice vaccinated with Gel + OVA, were too small or non-existent at the designated time point to retrieve sufficient material for analysis, tumours themselves were not analysed.

**Fig. 4:**
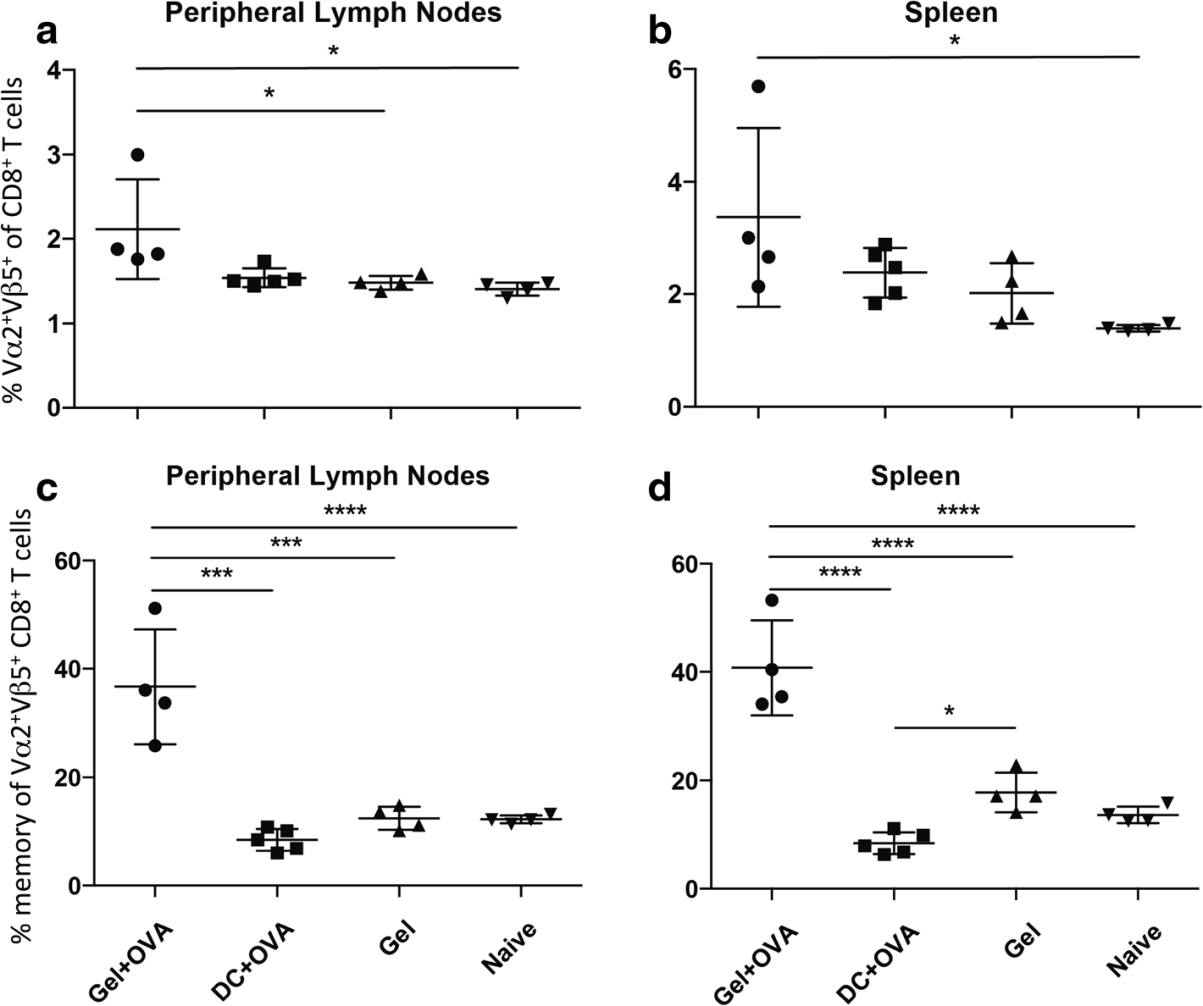
Vaccination with vaccine in a chitosan gel increases the frequency of OVA-specific CD8^+^ T cells post challenge with tumour. Mice received adoptive transfer of OT-I lymphocytes and were vaccinated with vaccine in a chitosan gel (Gel + OVA), the gel alone (Gel) or with vaccine pulsed-DC (DC + OVA) then challenged intracaecally with B16-OVA 30 days later. **a**, **b** Percentage of Vα2^+^Vβ5^+^ T cells of CD8^+^ T cells is shown in peripheral lymph nodes (**a**) and spleens (**b**) at day 21. **c**, **d** Percentage of CD122^+^CD44^+^ of Vα2^+^Vβ5^+^ CD8^+^ T cells is shown in peripheral lymph nodes (**c**) and spleens (**d**) at 21 days following challenge. Data are shown as mean ± standard deviation, *n* = 4–5, representative of two individual experiments. One-way ANOVA with Tukey post-hoc test was performed. *P < 0.05, **P < 0.01, ***P < 0.001, ****P < 0.0001

Previously, we have shown the Gel + OVA vaccination results in a higher frequency of antigen-specific as well as memory antigen-specific CD8+ T cells (prior to tumour challenge) than Gel only or DC-OVA vaccination [15]. To determine whether the higher cell number was consistent after tumour challenge and potentially mediating tumour protection, we measured the frequency of antigen-specific cells in mice following intra-caecal tumour injection. There was a significantly higher frequency of Vα2^+^Vβ5^+^ (OVA-specific) CD8^+^ T cells in the peripheral lymph nodes and spleens of mice vaccinated with Gel + OVA than with other vaccines (Fig. 4a, b). In the gut-associated lymphoid tissues, Peyer’s patches and mesenteric lymph nodes, no significant difference was seen in the frequency of Vα2^+^Vβ5^+^ CD8^+^ T cells seen at each site (data not shown). The frequency of CD122^+^CD44^+^ memory phenotype [21] cells in the OVA-specific CD8^+^ T cell population was significantly higher in those mice vaccinated with Gel + OVA compared to all other treatments in lymph nodes and spleen (Fig. 4c, d). The absolute number of cells was similar to the frequency of cells in each vaccination group (Additional file 3: Figure S3). Together, these data demonstrate that mice vaccinated with Gel + OVA acquired high frequencies of OVA-specific CD8^+^ effector and memory T cells compared to mice receiving other vaccines; similar to results seen in the melanoma model [15]. Taken together, these data indicate that chitosan gel vaccination increases the frequency of memory CD8+ T cells and this results in enhanced protection compared to other vaccines in both intra-caecal and subcutaneous tumour challenge.

### Protection by chitosan gel vaccine is associated with IFN-γ producing T cells

To determine the function of antigen-specific memory CD8^+^ T cells generated following vaccination, production of IFN-γ was measured in cells extracted from the spleens and Peyer’s patches of the vaccinated mice and from unvaccinated controls 3 weeks after intracaecal challenge with B16-OVA cells. To mimic studies of the immune response in human cancer [22], the extracted cells were incubated in the presence of PMA and ionomycin for 4 h and were analysed for expression of surface markers to detect CD4^+^ and CD8^+^ T cells, as well as Vα2^+^Vβ5^+^ to detect antigen-specific T cells, in conjunction with intracellular IFN-γ.

**Fig. 5:**
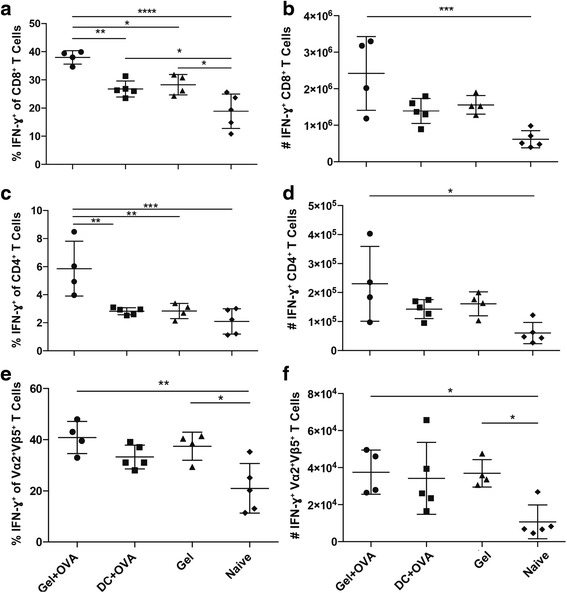
Vaccination with Gel + OVA increases cytokine production in the spleen. Mice received adoptive transfer of OT-I lymphocytes and were not vaccinated or were vaccinated with vaccine in a chitosan gel (Gel + OVA), the gel alone (Gel) or with vaccine pulsed-DC (DC + OVA) then challenged intracaecally with B16-OVA 30 days later. Percentage (**a**, **c**, **e**) and number (**b**, **d**, **f**) of IFN-γ^+^ CD8^+^ T cells (**a**, **b**), IFN-γ ^+^ CD4^+^ T cells (**c**, **d**), IFN-γ ^+^ Vα2Vβ5^+^ CD8^+^ T cells (**e**, **f**). All data shown are from spleen cells restimulated with PMA/ionomycin for 4 h, 21 days following challenge. Data are shown as mean ± standard deviation, *n* = 4–5, representative of two individual experiments. One-way ANOVA with Tukey post-hoc test was performed. *P < 0.05, **P < 0.01, ***P < 0.001, ****P < 0.0001

All immunised mice had a higher frequency of IFN-γ^+^ CD8^+^ T cells in the spleen, compared to unvaccinated mice (Fig. 5a, b), with the frequency of IFN-γ^+^ CD8^+^ T cells in mice immunised with Gel + OVA being the highest of vaccine groups. Within the CD4^+^ T cell compartment, a significant increase in the frequency of IFN-γ producing cells was found in cells isolated from the spleen of Gel + OVA vaccinated mice (Fig. 5c, d), and not the other groups, when compared to unvaccinated mice. A significant increase was seen in the frequency and number of IFN-γ producing Vα2^+^Vβ5^+^ CD8^+^ T cells in the spleens of mice vaccinated with Gel + OVA and a smaller, but still significant, increase in IFN-γ producing Vα2^+^Vβ5^+^ CD8^+^ T cells was also seen in the spleens of mice immunised with Gel alone (Fig. 5e, f). No difference was seen in IFN-γ-producing total memory T cell populations between groups (data not shown).

## Discussion

Colorectal cancer (CRC) is the third most common cancer in men and the second in women worldwide [1]. In order to evaluate new immune therapies in a preclinical setting, appropriate animal models are required. Here we have described an intra-caecal model of colorectal cancer in mice and shown that immune modulation of the systemic response can result in tumour protection associated with antigen specific T cells producing IFN-γ, consistent with the human disease [4, 22].

Interestingly we also demonstrated that biological location has an effect on the makeup of immune infiltrate into a tumour. It is well known that in many solid tumours infiltration of high numbers of T cells correlates with improved prognosis [4] and it has been reported that both B and T cells are important in human colorectal cancer [23]. Interestingly, in this study we found a difference in CD3+ T cell frequencies between intra-caecal and subcutaneous tumours, but no difference in the frequencies of CD4+ or CD8+ T cells. There are many subsets of T cells that may be reflected in this result, including pro-inflammatory “pathogenic” T cells [24], naturally occurring T cells that have down-regulated co-receptor expression [20, 25], NKT cells and MAIT cells [19], γδ T cells [26], and T cells in the context of immune deficiency [27]; all of which may be involved in anti-tumour immune responses, especially in the gut. Based on these results, further experiments should include an expanded panel of molecules to determine which of these CD3^+^ populations may have a significant effect on the local tumour immune response.

For mucosal immunity, priming of T cells by DCs in gut associated lymphoid tissues preferentially up-regulates mucosal homing receptors on those cells; hence the rationale for testing DC-OVA as a vaccine control. In the Peyer’s patches and mesenteric lymph nodes, CD103^+^ DCs cause up-regulation of CCR9 and α4β7 on lymphocytes, thus allowing them to migrate to gut mucosa [28–30]. Mucosal vaccines typically take advantage of this mechanism through administration at sites allowing antigen presentation to occur in gut associated lymphoid tissue. However, there are difficulties in developing oral peptide and protein based vaccines in terms of delivery of antigen in an immunogenic form to gut-associated lymphoid tissue. In the intra-caecal mouse model of cancer, the sustained release vaccine, but not a DC vaccine, provided protection. This protection was associated with an increased frequency of tumour specific T cells at peripheral lymphoid but not gut-associated lymphoid tissues. However, the absolute number of cells present in Peyer’s patches and mesenteric lymph nodes was low and it is possible that a later time-point is needed to detect differences. The frequency of tumour specific memory CD8^+^CD122^+^CD44^+^ T cells was significantly higher in mice vaccinated with the sustained release vaccine in peripheral lymphoid sites sampled consistent with our previous data prior to tumour challenge [15]. Here, we show that this increase is maintained after tumour challenge and is likely to be a correlate of protection. Further we show that the frequency of cytokine producing (IFN-γ) CD8^+^ and CD4^+^ T cells detected in the spleen was higher in Gel + OVA vaccinated mice than others, implying an IFNγ-mediated anti-tumour effect.

It is unclear if the tumours generated in this model are susceptible to systemic immunity or if gut-associated immune protection is required. Vaccination with the sustained release gel generated a population of antigen-specific CD8^+^ T cells in both Peyer’s patches and mesenteric lymph nodes, two gut-associated lymphoid sites, but this vaccine also stimulated stronger systemic responses than did the DC vaccine [15]. Therefore it is also possible that protection was conferred through effector memory T cells. While not mucosa specific, these cells are able to track through multiple tissues [31]. An effective effector memory T cell (T_em_) population generated through vaccination with a sustained release gel may provide protection in multiple different biological locations through the body.

The magnitude of the immune response generated by the DC vaccine was in general smaller as compared to the gel vaccine, similar to subcutaneous vaccination [15]. However the increased production of IFN-γ by OVA-specific CD8^+^ T cells in mice vaccinated with the gel compared to those vaccinated with the DC vaccine indicated that these cells could also have a functional difference. In preliminary experiments we also showed an increased frequency of IL-2^+^ OVA-specific CD8^+^ memory T cells in mice vaccinated with the gel vaccine compared to other vaccine groups (data not shown). It is possible that these cells may also have an increased capacity for proliferation and survival. This would allow for development of a more robust secondary recall population of CD8^+^ memory T cells upon tumour challenge.

These results also address a potential concern with the use of sustained release vaccines, namely that sustained release of vaccine may result in immune exhaustion rather than the development of effector or memory populations [32]. It is likely that the kinetics of release as well as the strength of the immune stimulation will impact on the type of response generated therefore caution should be taken when developing sustained release formulations in order to ensure that such parameters are optimised.

Interestingly the gel itself, in the absence of antigen, induced an increase in the frequency and number of antigen specific IFN-γ producing T cells in the spleens of mice challenged with OVA expressing tumour cells. Chitosan has been reported to have adjuvant activity [33, 34] and the gel, while not containing any antigen, was also loaded with the potent Th1 adjuvant Quil A [35]. The gel would have created an inflammatory depot that may have non-specifically boosted immune reactivity, much the same way the CpG have been used as a therapy to boost anti-tumor immunity [36]. Destruction of the tumour and release of OVA (a foreign antigen) by dying tumours would then have led to the development of some degree of antigen specific immunity. Mice immunised with the gel only did show some slight evidence of anti-tumor immunity. However as with the CpG studies, inclusion of a tumour antigen with the therapy increases the efficacy of the response [36].

The B16 cell line used for intracaecal challenge is a melanoma cell line. While this allowed for direct comparison to subcutaneous B16 challenge ref, in this mouse model of colorectal cancer it may be beneficial to use a mouse colorectal cancer cell line. Further experiments using the CT26 colorectal cancer cell line expressing an endogenous peptide [37, 38] or a MC38 colorectal cancer cell line expressing OVA [39] may provide more insight into the disease by providing more accuracy to the model system.

## Conclusions

In a clinical setting, cancer is generally treated therapeutically. In this research, mice were vaccinated prophylactically in order to assess the memory populations of T cells generated. Although vaccination with the sustained release proved successful in this setting, further work is required to find whether the same is true when used as a therapy as shown in a subcutaneous model [15]. If this treatment were to be effective, it would further implicate chitosan gel as a potential anti-cancer vaccine strategy. The ability of vaccination with a sustained release vaccine to work therapeutically would rely on the ability of the activated immune cells to overcome the immunosuppressive effect of the tumour. It is likely this approach would be combined with others targeted at overcoming tumour induced immune suppression in order to achieve maximal therapeutic benefit.

## Additional files

Additional file 1: Figure S1. Flow cytometry gating for identification of tumour immune cell infiltrates. Singlets were identified by gating with FSC- H versus FSC-A then SSC-H versus SSC-A. Live cells had low levels of Live/Dead dye. Lymphocytes and large cells were identified based on SSC-A and FSC-A. F4/80 expression was used to designate macrophages within the large cell gate and CD11c was used as an identifying marker of dendritic cells. CD19 was used to identify B cells within the lymphocyte gate and CD3 was used to identify T cells. CD8^+^ and CD4^+^ T cells were identified within the CD3 gate. (PDF 1568 kb)
Additional file 2: Figure S2. Flow cytometry gating for identification of cytokine producing cells following vaccination and subcutaneous tumour challenge. Singlets were identified by gating with FSC-H versus FSC-A then SSC-H versus SSC-A. Live cells had low levels of Live/Dead dye and lymphocytes were identified based on SSC-A and FSC-A. CD8^+^ T cells were those that expressed CD8 and CD4^+^ T cells were those expressing CD4. Vα2, Vβ5 double positive cells were identified within the CD8 gate and those expressing CD122 and CD44 were defined as memory cells. IFN-γ production was analysed in CD8^+^ T cells, CD4^+^ T cells, Vα2^+^Vβ5^+^ cells and CD122^+^CD44^+^ memory cells. (PDF 1292 kb)
Additional file 3: Figure S3. Vaccination with vaccine in a chitosan gel increases the absolute cell number of OVA-specific CD8^+^ T cells post challenge with tumour. Mice received adoptive transfer of OT-I lymphocytes and were vaccinated with vaccine in a chitosan gel (Gel + OVA), the gel alone (Gel) or with vaccine pulsed-DC (DC + OVA) then challenged intra-caecally with B16-OVA 30 days later. (A, B) Number of Vα2^+^Vβ5^+^ T cells of CD8^+^ T cells is shown in peripheral lymph nodes (A) and spleens (B) at day 21. (C, D) Number of CD122^+^CD44^+^ of Vα2^+^Vβ5^+^ CD8^+^ T cells is shown in peripheral lymph nodes (C) and spleens (D) at 21 days following challenge. Data are shown as mean ± standard deviation, *n* = 4–5, representative of two individual experiments. One-way ANOVA with Tukey post-hoc test was performed. ***P* < 0.01. (EPS 1138 kb)
