# Supplementary figures and images for: Chitosan gel vaccine protects against tumour growth in an intracaecal mouse model of cancer by modulating systemic immune responses
Source: BMC Immunol. 2016 Oct 18;17:39. doi: 10.1186/s12865-016-0178-4 (PMC5069793; doi:10.1186/s12865-016-0178-4)

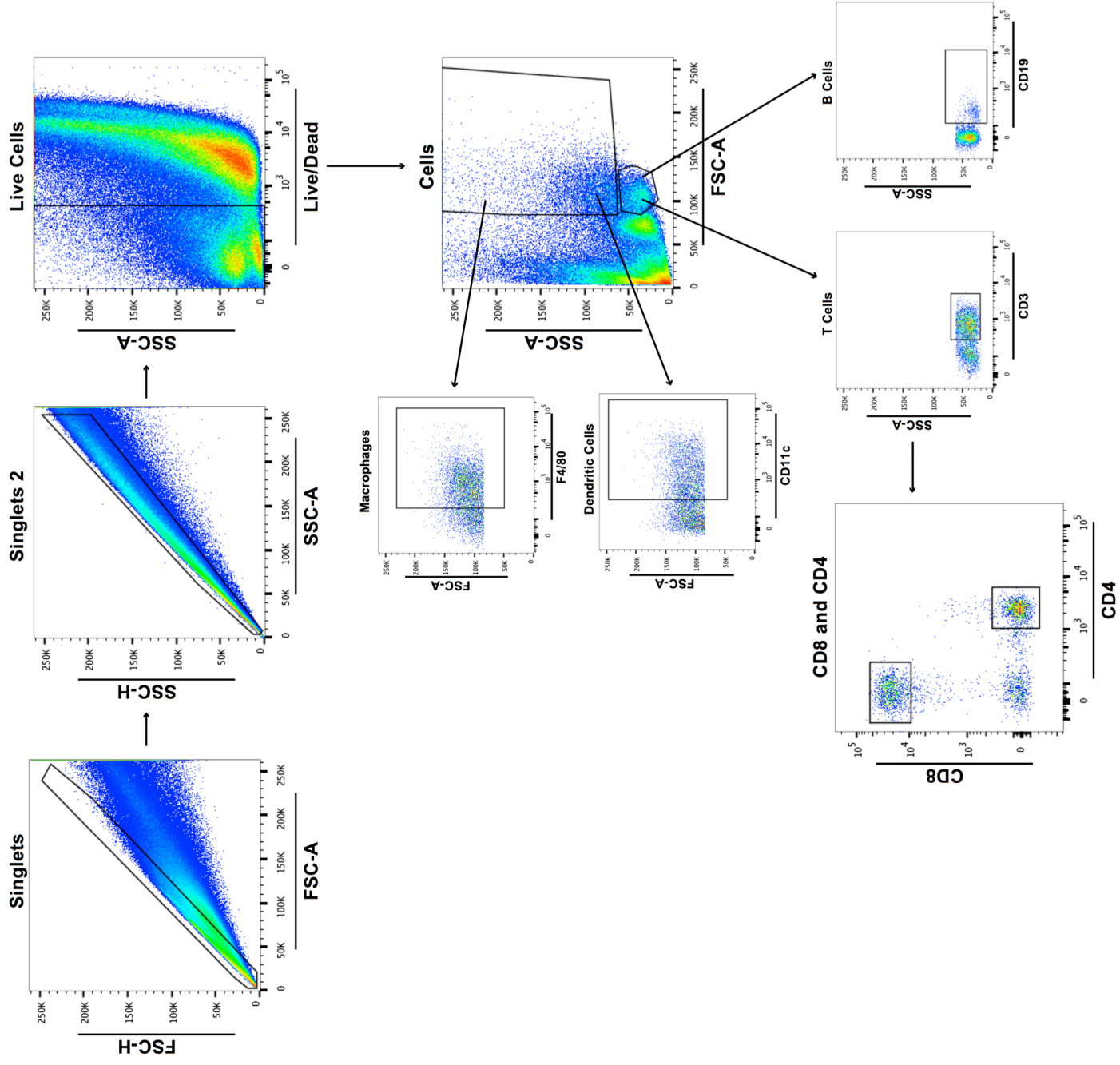

Highton et al  
 SUPPLEMENTARY  
 FIGURE 1

Supplement: Additional file 1: Figure S1. — Flow cytometry gating for identification of tumour immune cell infiltrates. Singlets were identified by gating with FSC- H versus FSC-A then SSC-H versus SSC-A. Live cells had low levels of Live/Dead dye. Lymphocytes and large cells were identified based on SSC-A and FSC-A. F4/80 expression was used to designate macrophages within the large cell gate and CD11c was used as an identifying marker of dendritic cells. CD19 was used to identify B cells within the lymphocyte gate and CD3 was used to identify T cells. CD8+ and CD4+ T cells were identified within the CD3 gate. (PDF 1568 kb) [file 12865_2016_178_MOESM1_ESM.pdf]

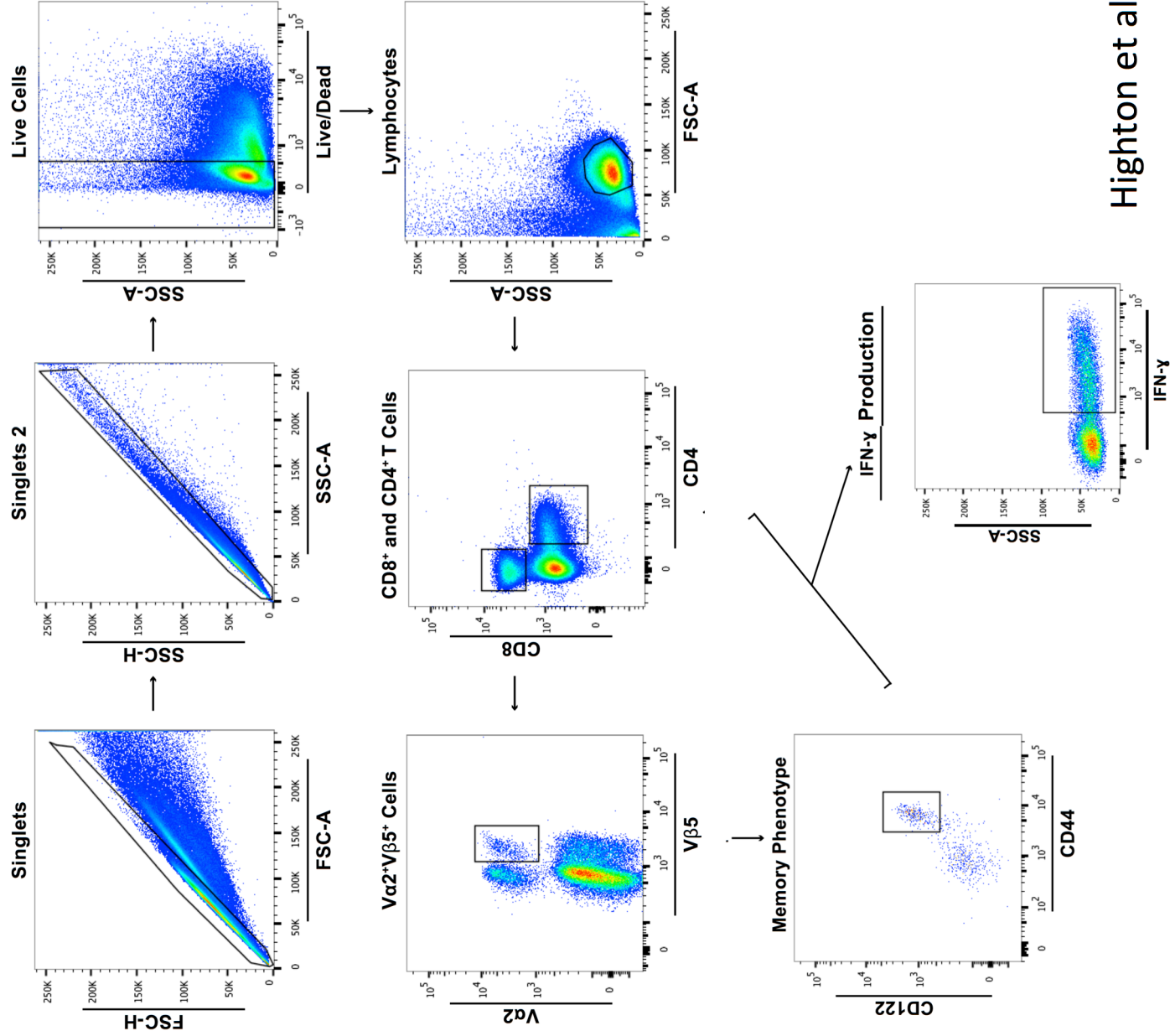

Highton et al SUPPLEMENTARY FIGURE 2

Supplement: Additional file 2: Figure S2. — Flow cytometry gating for identification of cytokine producing cells following vaccination and subcutaneous tumour challenge. Singlets were identified by gating with FSC-H versus FSC-A then SSC-H versus SSC-A. Live cells had low levels of Live/Dead dye and lymphocytes were identified based on SSC-A and FSC-A. CD8+ T cells were those that expressed CD8 and CD4+ T cells were those expressing CD4. Vα2, Vβ5 double positive cells were identified within the CD8 gate and those expressing CD122 and CD44 were defined as memory cells. IFN-γ production was analysed in CD8+ T cells, CD4+ T cells, Vα2+Vβ5+ cells and CD122+CD44+ memory cells. (PDF 1292 kb) [file 12865_2016_178_MOESM2_ESM.pdf]
